# Galaxy mothur Toolset (GmT): a user-friendly application for 16S rRNA gene sequencing analysis using mothur

**DOI:** 10.1093/gigascience/giy166

**Published:** 2018-02-28

**Authors:** Saskia D Hiltemann, Stefan A Boers, Peter J van der Spek, Ruud Jansen, John P Hays, Andrew P Stubbs

**Affiliations:** 1 Erasmus University Medical Center Rotterdam, Department of Pathology, Bioinformatics group, Wytemaweg 80, 3015 CN, Rotterdam, The Netherlands; 2 Erasmus University Medical Center Rotterdam, Department of Medical Microbiology and Infectious Diseases, Dr. Molewaterplein 40, 3015 GD, Rotterdam, The Netherlands; 3 Regional Laboratory of Public Health Kennemerland, Department of Molecular Biology, Boerhaavelaan 26, 2035 RC, Haarlem, The Netherlands

**Keywords:** microbial classification, 16S rRNA gene sequence analysis, mothur

## Abstract

**Background:**

The determination of microbial communities using the mothur tool suite (https://www.mothur.org) is well established. However, mothur requires bioinformatics-based proficiency in order to perform calculations via the command-line. Galaxy is a project dedicated to providing a user-friendly web interface for such command-line tools (https://galaxyproject.org/).

**Results:**

We have integrated the full set of 125+ mothur tools into Galaxy as the Galaxy mothur Toolset (GmT) and provided a set of workflows to perform end-to-end 16S rRNA gene analyses and integrate with third-party visualization and reporting tools. We demonstrate the utility of GmT by analyzing the mothur MiSeq standard operating procedure (SOP) dataset (https://www.mothur.org/wiki/MiSeq_SOP).

**Conclusions:**

GmT is available from the Galaxy Tool Shed, and a workflow definition file and full Galaxy training manual for the mothur SOP have been created. A Docker image with a fully configured GmT Galaxy is also available.

## Findings

### Introduction

A 16S rRNA gene profiling analysis can be achieved using an extensive array of sophisticated software including mothur [1], QIIME [2], MG-RAST [3], and many more [4]. While some of these applications have a graphical user interface to provide access to these technologies for the research scientist, their use remains complex for non-bioinformaticians. In this respect, the Galaxy project [5] was developed in order to simplify the use of complex command-line software tools. Galaxy offers extensive support for both 16S rRNA gene-based and broader metagenomic analyses, with more than 100 tools in the metagenomics section of the Galaxy tool shed, including QIIME [2], Krona [6], PyNAST [7], PICRUSt [8], Kraken [9], MetaPhlAn2 [10], HUMAnN2 [11], PrinSEQ [12], Nonpareil [13], Vegan [14], and many more.

mothur is an open-source application that was designed as a single piece of software capable of analyzing and comparing microbial communities from 16S rRNA gene data derived from next-generation sequencing (NGS). The creators of mothur did not only provide an extensive set of tools but also a collection of standard operating procedures (SOPs) that detail the recommended analytical protocol for different types of input data.

The latest version of mothur consists of more than 125 components, lending it great flexibility but, at the same time, great complexity. To address this challenge, we have integrated the full set of 125+ mothur components into Galaxy that are collectively called the "Galaxy mothur Toolset" (GmT). To simplify usage of GmT, we provide the full workflow definition files, usage of which shields the end user from the full complexities of the analysis. By simultaneously providing access to all the individual components present in mothur as separate tools, expert users and bioinformaticians retain the ability to utilize the full flexibility of mothur by creating custom workflows or by modifying or extending our workflows to fit their use-case.

GmT also leverages Galaxy’s collections framework to enable easy analysis of large numbers (many thousands) of samples at once. Many mothur components support parallel computing, and the Galaxy tools will utilize the maximum amount of processing power allotted to them by the instance administrator (Supplementary data S2). As part of GmT, datatypes were also contributed to the Galaxy core codebase to facilitate the handling of mothur-specific datatypes within Galaxy. Furthermore, a Galaxy data manager was also created for the automatic installation and configuration of reference datasets utilized by the mothur tool suite. Last, a Galaxy interactive environment (GIE) [15] for Phinch [16] was also developed [17].

GmT includes tools to produce standard file formats, such as the Biological Observation Matrix (BIOM) format [18], to facilitate interoperability with these downstream analysis components. Where no clear file standards exist, GmT provides custom tools for conversion of mothur datatypes to other tools (e.g., the taxonomy-2-krona tool). This allows for integration with third-party tools such as PICRUSt for prediction of functional content or visualization tools such as Phinch, Krona, and certain QIIME components (Supplementary data S1). The mothur tools also natively support incorporation of some third-party analysis tools such as UCHIME and ChimeraSlayer for chimera detection or VSEARCH for clustering, which are also available in GmT.

The Galaxy Training Network (GTN) [19] is a network of people and groups that present Galaxy and Galaxy-based training around the world. The GTN has created a central repository [20] for Galaxy training materials. In order to further facilitate the use of GmT to end users, we have contributed training materials to the GTN that illustrate how to run mothur’s MiSeq SOP within Galaxy [21]. This work has also been incorporated in a larger-scale framework to easily and quickly explore microbiota data in a reproducible and transparent environment [22].

### Purpose of this work

The work performed and described in this technical note has four objectives. First is to provide end users and bioinformaticians with easy access to all the mothur tools as the GmT. Second is to provide open-access online training material to demonstrate/complete the mothur SOP in Galaxy. Third is to deliver an end-to-end workflow for the mothur SOP in Galaxy that is available for upload to any Galaxy that has the GmT installed. Fourth is to provide a summarization of results in a web report using the iReport Galaxy tool [23]. Our aim is to provide 16S rRNA gene NGS analysis tools and awareness on how to use them in a format that supports FAIR data principles [24].

### Worked Example

**Figure 1:**
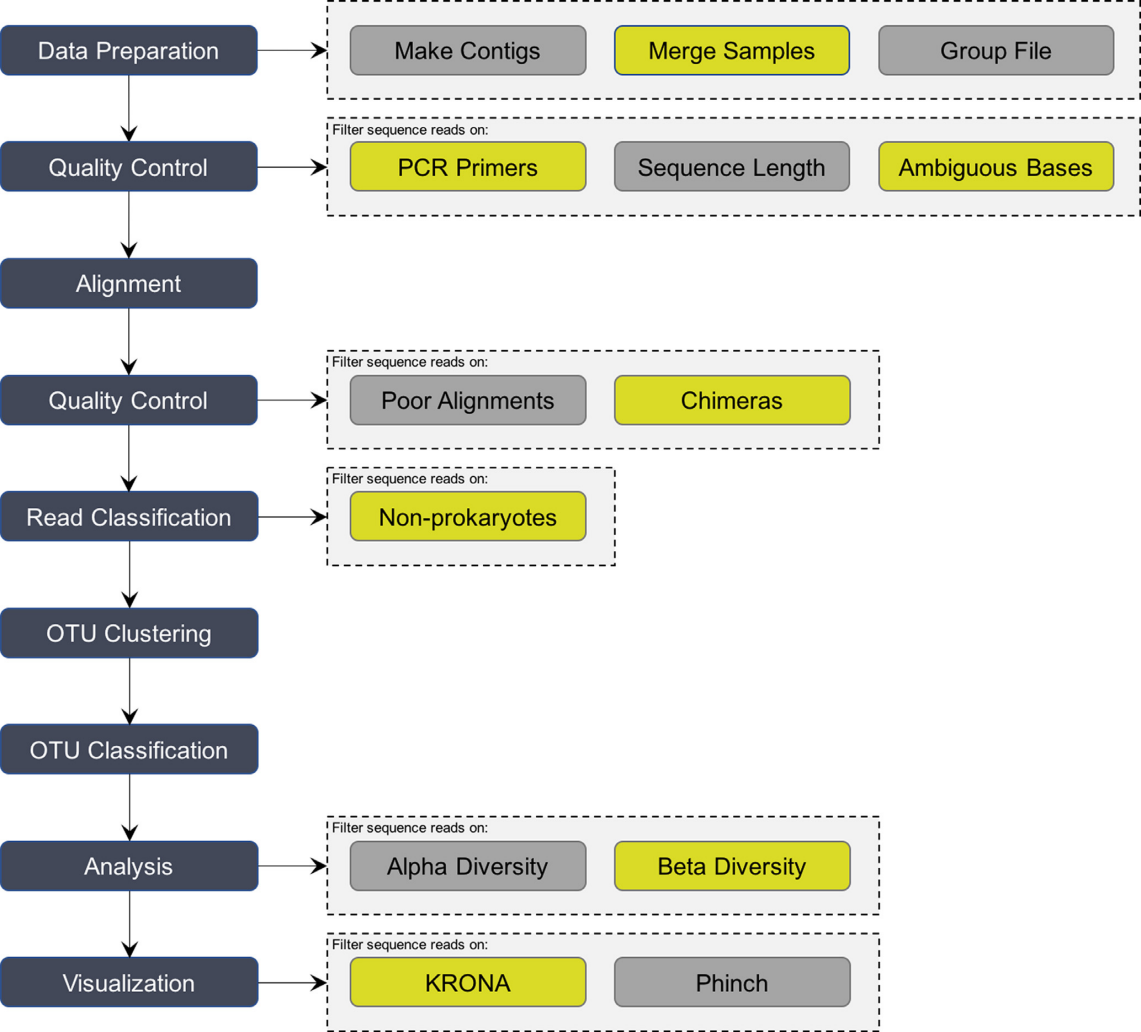
Conceptual view of the GmT mothur MiSeq SOP pipeline.

To illustrate the utility of our toolkit, we present results on example data below. GmT is designed to take short-read 16S rRNA gene NGS data as input and to output a dynamic web report for prokaryotic taxonomical classification using the Galaxy platform. A GmT workflow follows essentially a four-step process:

1. **Data upload**. The Galaxy platform provides the users with standard data upload functionality for single and multi-sample datasets.
2. **Collection creation**. For multi-sample and/or paired-end datasets, a Galaxy collection must be created in the Galaxy interface. Here, datasets can also be assigned to groups. Galaxy will make intelligent suggestions for pairings of datasets based on the file names.
3. **16S rRNA gene analysis**. mothur has been wrapped as a tool suite in Galaxy. Required steps included for a full "end-to-end" 16S rRNA gene sequencing analysis consist of read-pair merging (mothur command: make.contigs), trimming of primer sequences (trim.seqs), additional quality control (screen.seqs), alignment of sequences to a (customized) reference alignment (align.seqs, screen.seqs), removal of chimeric sequences (chimera.uchime), classifying sequences using a Bayesian classifier in combination with a reference database such as SILVA or GreenGenes (classify.seqs), and clustering of sequences into operational taxonomic units (OTUs) at a predefined percentage, usually 97%, of similarity (dist.seqs, cluster, and classify.otu) (Fig. 1).
4. **Experimental summary and reporting**. iReport in combination with Krona is used to deliver an HTML report in Galaxy [6]. The iReport consists of multiple tabs to group results topically (e.g., taxonomy, rarefaction, diversity, quality control) and is highly customizable and easily tailored to an end user’s specific use-case. The entire report may be downloaded from the Galaxy interface to be viewed or shared offline.
  To compare the output from a single experiment or across multiple experiments, we utilized Phinch [16], a dynamic web application that uses BIOM-formatted files to explore and analyze biological patterns in 16S rRNA gene NGS datasets.

## Methods

### Handling large datasets

Large-scale analyses have become the norm in the field, both large in disk space as in the number of files, and this can pose a challenge for analysis. For large files, Galaxy offers the option of uploading via FTP rather than web transfer. The introduction of the concept of "collections" in Galaxy has enabled users to analyze datasets consisting of a large number of files (>100 K) as easily as they would a single file.

### Galaxy mothur toolset

Many mothur components support parallelization, and our Galaxy wrappers will run these components with the maximum number of CPUs allotted to them by the Galaxy administrator. In order to diagnose potential failures, Galaxy outputs the full standard and error logs, which the users can inspect. Furthermore, we have contributed mothur datatype definitions to the Galaxy core code, meaning that the users will be protected from inputting the wrong datasets and thus reduce the number of errors they will make with the tools. All tools in GmT use only conda dependencies, making their installation in Galaxy a painless experience that requires nothing more than a single press of a button.

The mothur tool wrappers have been submitted to the Intergalactic Utilities Commission (IUC) tool repository [25] and are available from the Galaxy Tool Shed [26]. The IUC is a group of community members dedicated to developing and upholding Galaxy tool development best practices and guidelines. Thus, by contributing our tools to this repo, we ensure that the tools will be well maintained. A metagenomics Galaxy flavour [27] that contains all components presented here is available. The full mothur suite has also been installed to Galaxy’s main server [28].

### Krona visualization

Krona [6] is a data viewer that provides the ability to interactively explore hierarchical data. A Galaxy Krona wrapper that works directly on mothur data formats was developed for this project.

### Phinch visualization

Galaxy offers integration with Phinch [16] BIOM format viewer in two ways: as a GIE developed in the context of this project [17] and, more recently, as an external display application hosted by the Galaxy team.

### iReport summarization

**Figure 2:**
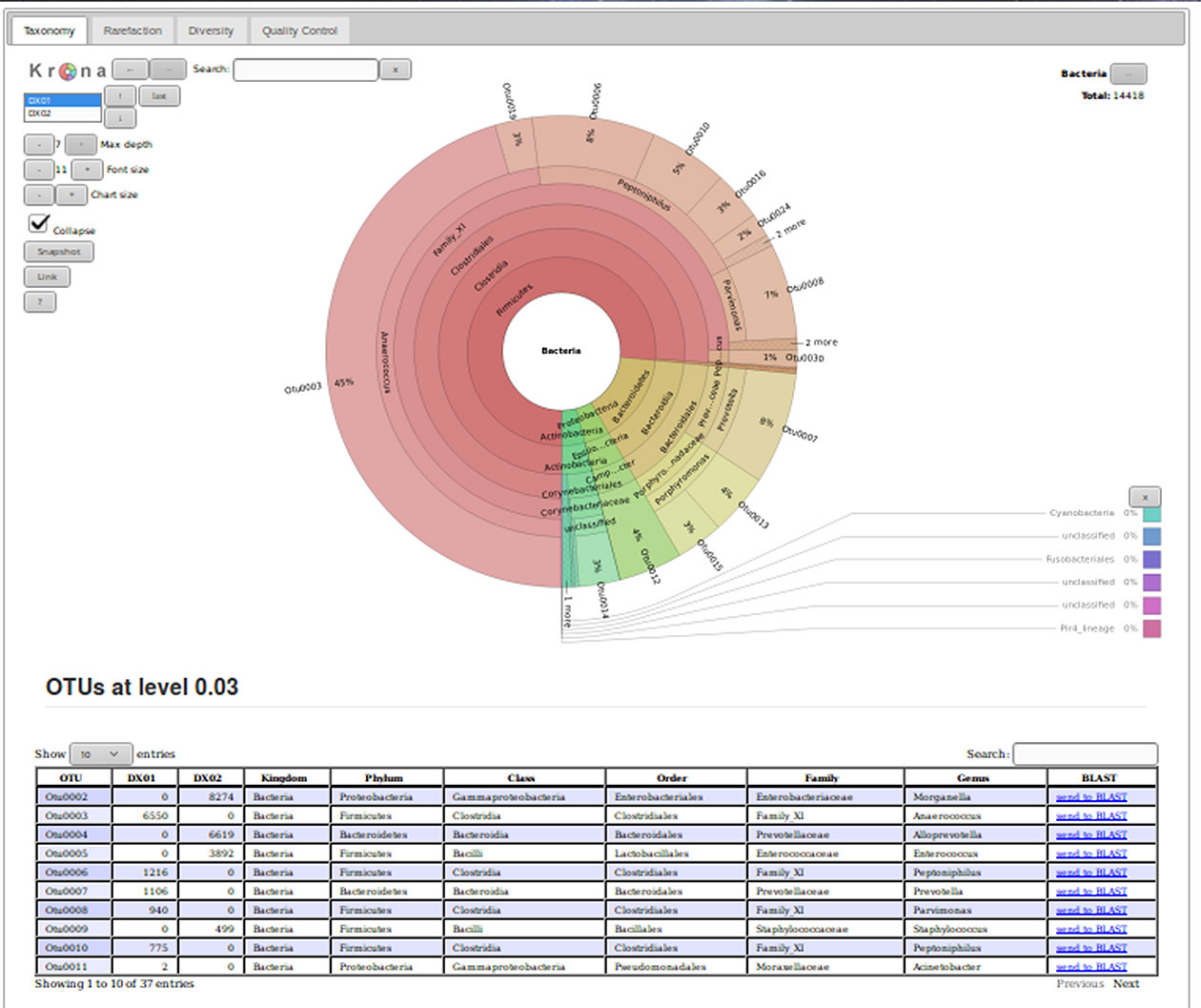
Example iReport. This web report contains the interactive Krona visualization, the (multi-sample) operational taxonomic unit table, rarefaction plots, diversity calculations, differential abundance analysis, and an extensive overview of the quality-control measurements taken during the analysis. iReports are highly customizable and can be easily tailored to fit specific use-cases and end-user needs.

To facilitate the evaluation of 16S rRNA gene sequencing analysis results, integration with the iReport [23] tool is also provided. This tool creates a web report to present the analysis results in an organized fashion and provides links to external resources such as Basic Local Alignment Search Tool searches (Fig. 2).

## Availability of source code and requirements

- Project name: Galaxy mothur Toolset (GMT)
- Project home page: https://github.com/erasmusmc-bioinformatics/galaxy-mothur-toolset
- Toolshed repository: https://toolshed.g2.bx.psu.edu/view/iuc/suite_mothur/768c2e48b706
- Training manual: https://galaxyproject.github.io/training-material
- GmT Docker image: https://quay.io/shiltemann/galaxy-mothur-toolset:16.07
- Galaxy Metagenomics Docker Flavour (Docker): https://quay.io/repository/shiltemann/galaxy-metagenomics, https://github.com/shiltemann/galaxy-metagenomics
- Phinch interactive environment: https://github.com/shiltemann/phinch-galaxy-ie
- Operating system(s): Unix (Platform independent with Docker)
- License: GNU GPL v3

## Availability of supporting data

The data presented here to illustrate our work are the same data used in the training manual and is available from Zenodo [29]. Code snapshots, benchmarking data, and example report files are also available in the GigaScience GigaDB repository [30].

## Abbreviations

BIOM: Biological Observation Matrix; GIE, Galaxy interactive environment; GmT, Galaxy mothur Toolset; GTN, Galaxy Training Network; IUC: Intergalactic Utilities Commission; NGS: next-generation sequencing; OUT: operational taxonomic unit; SOP, standard operating procedure.

## Competing Interests

The authors declare that they have no competing interests.

## Funding

This work has received funding from the European Union’s Seventh Framework Programme for Health under grant agreement 602860 (TAILORED-Treatment; http://www.tailored-treatment.eu) and from the Eurostars Programme under grant agreement E! 10959 iKnowIT.

## Author Contributions

S.H. developed the Galaxy tool wrappers and Phinch interactive environment. S.B. validated the analysis pipelines. All authors contributed to the manuscript text and approve its contents.

## Supplementary Material

GIGA-D-18-00110.pdf

GIGA-D-18-00110_R1.pdf

GIGA-D-18-00110_R2.pdf

Response_to_Reviewer_Comments_Original_Submission.pdf

Response_to_Reviewer_Comments_Revision_1.pdf

Reviewer_1_Report_original_submission -- Hiroshi Mori 4/8/2018 Reviewed

Reviewer_2_Report_original_submission -- Xianfeng Chen 4/16/2018 Reviewed

Supplemental Files
