# Supplementary material for: Galaxy mothur Toolset (GmT): a user-friendly application for 16S rRNA gene sequencing analysis using mothur
Source: Gigascience. 2018 Feb 28;8(2):giy166. doi: 10.1093/gigascience/giy166 (PMC6377400; doi:10.1093/gigascience/giy166)
Supplement: GIGA-D-18-00110.pdf [file giy166_giga-d-18-00110.pdf]

GigaScience

Galaxy mothur Toolset (GmT): a user-friendly application for 16S rRNA gene sequencing analysis using mothur.

--Manuscript Draft--

**Manuscript Number:**	GIGA-D-18-00110
**Full Title:**	Galaxy mothur Toolset (GmT): a user-friendly application for 16S rRNA gene sequencing analysis using mothur.
**Article Type:**	Technical Note
**Funding Information:**	FP7 Health (R.Jansen@streeklabhaarlem.nl) — Dr Ruud Jansen
**Abstract:**	Background<br>The determination of microbial communities using the mothur tool suite (https://www.mothur.org) is well established. However, mothur requires bioinformatics-based proficiency in order to perform calculations via the command-line. Galaxy is a project dedicated to providing a user-friendly web interface for such command-line tools (https://galaxyproject.org/).<br><br>Results<br>We have integrated the full set of 125+ mothur tools into Galaxy as the Galaxy mothur Toolset (GmT) and provided a set of workflows to perform end-to-end 16S rRNA gene analyses and integrate with third-party visualization and reporting tools. We demonstrate the utility of GmT by analysing the mothur MiSeq standard operating procedure (SOP) data set (https://www.mothur.org/wiki/MiSeq_SOP).<br><br>Conclusions<br>GmT is available from the Galaxy Tool Shed, and a workflow definition file and full Galaxy training manual for the mothur SOP have been created. A Docker image with a fully configured GmT Galaxy is also available.
**Corresponding Author:**	Saskia Hiltemann<br><br>NETHERLANDS
**Corresponding Author Secondary Information:**	
**Corresponding Author's Institution:**	
**Corresponding Author's Secondary Institution:**	
**First Author:**	Saskia Hiltemann
**First Author Secondary Information:**	
**Order of Authors:**	Saskia Hiltemann<br>Stefan Boers<br>Peter van der Spek<br>Ruud Jansen<br>John Hays<br>Andrew Stubbs
**Order of Authors Secondary Information:**	
**Additional Information:**	
**Question**	**Response**
Are you submitting this manuscript to a special series or article collection?	No

Powered by Editorial Manager® and ProduXion Manager® from Aries Systems Corporation

**Experimental design and statistics**<br><br>Full details of the experimental design and statistical methods used should be given in the Methods section, as detailed in our Minimum Standards Reporting Checklist. Information essential to interpreting the data presented should be made available in the figure legends.<br><br>Have you included all the information requested in your manuscript?	Yes
**Resources**<br><br>A description of all resources used, including antibodies, cell lines, animals and software tools, with enough information to allow them to be uniquely identified, should be included in the Methods section. Authors are strongly encouraged to cite Research Resource Identifiers (RRIDs) for antibodies, model organisms and tools, where possible.<br><br>Have you included the information requested as detailed in our Minimum Standards Reporting Checklist?	Yes
**Availability of data and materials**<br><br>All datasets and code on which the conclusions of the paper rely must be either included in your submission or deposited in publicly available repositories (where available and ethically appropriate), referencing such data using a unique identifier in the references and in the "Availability of Data and Materials" section of your manuscript.<br><br>Have you have met the above requirement as detailed in our Minimum Standards Reporting Checklist?	Yes

Manuscript
Click here to download Manuscript main.tex

Placeholder for OUP logo oup.pdf

Placeholder for journal logo gigascience-logo.pdf

GigaScience, 2017, 1–4

doi: xx.xxxx/xxxx
Manuscript in Preparation
Technical Note

TECHNICAL NOTE

Galaxy mothur Toolset (GmT): a user-friendly application for 16S rRNA gene sequencing analysis using mothur.

Saskia D. Hiltemann[1,*,†], Stefan A. Boers[2,†], Peter J. van der Spek[1], Ruud Jansen[3], John P. Hays[2] and Andrew P. Stubbs[1]

[1]Erasmus University Medical Center Rotterdam, Department of Bioinformatics and [2]Erasmus University Medical Center Rotterdam, Department of Medical Microbiology and Infectious Diseases and [3]Regional Laboratory of Public Health Kennemerland, Department of Molecular Biology

[*] Corresponding author: s.hiltemann@erasmusmc.com
[†]Contributed equally.

Abstract

Background The determination of microbial communities using the mothur tool suite (https://www.mothur.org) is well established. However, mothur requires bioinformatics-based proficiency in order to perform calculations via the command-line. Galaxy is a project dedicated to providing a user-friendly web interface for such command-line tools (https://galaxyproject.org/).
Results We have integrated the full set of 125+ mothur tools into Galaxy as the Galaxy mothur Toolset (GmT) and provided a set of workflows to perform end-to-end 16S rRNA gene analyses and integrate with third-party visualization and reporting tools. We demonstrate the utility of GmT by analysing the mothur MiSeq standard operating procedure (SOP) data set (https://www.mothur.org/wiki/MiSeq_SOP).
Conclusions GmT is available from the Galaxy Tool Shed, and a workflow definition file and full Galaxy training manual for the mothur SOP have been created. A Docker image with a fully configured GmT Galaxy is also available.

Key words: Microbial classification; 16S rRNA gene sequence analysis; mothur

Findings

Introduction

16S rRNA gene profiling analysis can be achieved using an extensive array of sophisticated software including mothur [1], QIIME [2], MG-RAST [3], and many more [4]. Whilst some of these applications have a graphical user interface (GUI) to provide access to these technologies for the research scientist, their use remains complex for non-bioinformaticians. In this respect, the Galaxy project [5] was developed in order to simplify the use of complex command-line software tools. Galaxy offers extensive support for both 16S rRNA gene-based and broader metagenomic analyses, with over 100 tools in the metagenomics section of the Galaxy tool shed, including QIIME [2], KRONA [6], PyNAST [7], PICRUSt [8], Kraken [9], MetaPhlAn2 [10], HUMAnN2 [11], PrinSEQ [12], Nonpareil [13], Vegan [14], and many more.

mothur is an open-source application that was designed as a single piece of software capable of analysing and comparing microbial communities from 16S rRNA gene data derived from next-generation sequencing (NGS). The creators of mothur did not only provide an extensive set of tools, but also a collection of standard operating procedures (SOPs) that detail the recommended analytical protocol for different types of input data.

The latest version of mothur consists of over 125 compo-

Compiled on: March 28, 2018.
Draft manuscript prepared by the author.

Key Points

- GmT provides a user-friendly interface to mothur by implementing mothur software in Galaxy
- A Galaxy workflow and full training manual for the mothur SOP are provided
- GmT provides integration with third-party visualization and reporting tools

nents, lending it great flexibility, but at the same time, great complexity. To address this challenge, we have integrated the full set of 125+ mothur components into Galaxy that are collectively called the 'Galaxy mothur Toolset (GmT)'. To simplify usage of GmT we provide the full workflow definition files, usage of which shields the end-user from the full complexities of the analysis. By simultaneously providing access to all the individual components present in mothur as separate tools, expert users and bioinformaticians retain the ability to utilize the full flexibility of mothur by creating custom workflows or by modifying or extending our workflows to fit their use-case.

GmT also leverages Galaxy's collections framework to enable easy analysis of large numbers (many thousands) of samples at once. Many mothur components support parallel computing, and the Galaxy tools will utilize the maximum amount of processing power alloted to them by the instance administrator. As part of GmT, datatypes were also contributed to the Galaxy core codebase to facilitate the handling of mothur-specific datatypes within Galaxy. Furthermore, a Galaxy data manager was also created for the automatic installation and configuration of reference datasets utilized by the mothur tool suite. And lastly, a Galaxy Interactive environment (GIE) [15] for Phinch [16] was also developed [17].

GmT includes tools to produce standard file formats, such as the BIOM format [18] to facilitate interoperability with these downstream analysis components. Where no clear file standards exists, GmT provides custom tools for conversion of mothur datatypes to other tools (e.g. the taxonomy-2-krona tool). This allows for integration with third party tools such as PICRUSt for prediction of functional content, or visualisation tools such as Phinch, KRONA, and certain QIIME components. The mothur tools also natively support incorporation of some 3rd party analysis tools, such as UCHIME and ChimeraSlayer for chimera detection or VSEARCH for clustering, which are also available in GmT.

The Galaxy Training Network (GTN) is a network of people and groups that present Galaxy and Galaxy-based training around the world. The GTN has created a central repository [19] for Galaxy training materials. In order to further facilitate the use of GmT to end-users, we have contributed training materials to the GTN that illustrate how to run mothur's MiSeq SOP within Galaxy [20]. This work has also been incorporated in a larger-scale framework to easily and quickly explore microbiota data in a reproducible and transparent environment [21].

Purpose of this work

The work performed and described in this technical note has four objectives. First to provide end-users and bioinformaticians with easy access to all the mothur tools as the GmT. Second is to provide open-access online training material to demonstrate/complete the mothur SOP in Galaxy. Third is to deliver an end-to-end workflow for the mothur SOP in Galaxy that is available for upload to any Galaxy that has the GmT installed. Fourth is to provide a summarization of results in a web report using the iReport Galaxy tool [22]. Our aim is to provide 16S rRNA gene NGS analysis tools and awareness on how to use them in a format that supports FAIR data principles [23].

Worked Example

To illustrate the utility of our toolkit, we present results on example data below. GmT is designed to take short-read 16S rRNA gene NGS data as input and to output a dynamic web report for prokaryotic taxonomical classification using the Galaxy platform. A GmT workflow follows essentially a four-step process:

i. **Data upload**. The Galaxy platform provides the users with standard data upload functionality for single and multi-sample datasets.
ii. **Collection Creation**. For multi-sample and/or paired-end datasets a Galaxy collection must be created in the Galaxy interface. Here datasets can also be assigned to groups. Galaxy will make intelligent suggestions for pairings of datasets based on the file names.
iii. **16S rRNA gene analysis**. mothur has been wrapped as a tool suite in Galaxy. Required steps included for a full 'end-to-end' 16S rRNA gene sequencing analysis consist of read-pair merging (mothur command: make.contigs), trimming of primer sequences (trim.seqs), additional quality control (screen.seqs), alignment of sequences to a (customized) reference alignment (align.seqs, screen.seqs), removal of chimeric sequences (chimera.uchime), classifying sequences using a Bayesian classifier in combination with a reference database such as SILVA or GreenGenes (classify.seqs), and clustering of sequences into operational taxonomic units (OTUs) at a predefined percentage - usually 97 percent - of similarity (dist.seqs, cluster, and classify.otu) (Figure 1).
iv. **Experimental Summary and Reporting**. iReport in combination with KRONA is used to deliver an HTML report in Galaxy [6]. The iReport consists of multiple tabs to group results topically (e.g. taxonomy, rarefaction, diversity, quality control) and is highly customizable and easily tailored to an end-user's specific use-case. The entire report may be downloaded from the Galaxy interface to be viewed or shared offline.
To compare the output from a single experiment or across multiple experiments we utilized Phinch [16], a dynamic web application which uses BIOM-formatted files to explore and analyse biological patterns in 16S rRNA gene NGS datasets.

Methods

Handling large datasets

Large-scale analyses have become the norm in the field, both large in disk space as in the number of files, and this can pose a challenge for analysis. For large files, Galaxy offers the option of uploading via FTP rather than web transfer. The introduction of the concept of "collections" in Galaxy has enabled users to analyze datasets consisting of a large number of files (>100K) as easily as they would a single file.

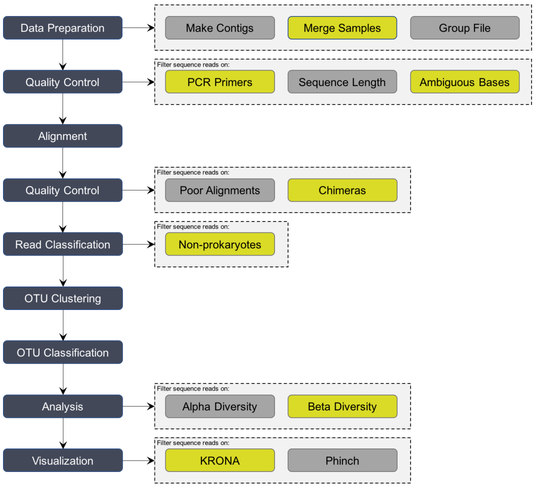

Figure 1. Conceptual view of the GmT mothur MiSeq SOP pipeline.

Galaxy mothur Toolset

Many mothur components support parallelization, and our Galaxy wrappers will run these components with the maximum number of CPUs allotted to them by the Galaxy administrator. In order to diagnose potential failures, Galaxy outputs the full standard and error logs, which the users can inspect. Furthermore, we have contributed mothur datatype definitions to the Galaxy core code, meaning that the users will be protected from inputting the wrong datasets and thus reduce the number of errors they will make with the tools. All tools in GmT use only conda dependencies, making their installation in Galaxy a painless experience that requires nothing more than a single press of a button.

The mothur tool wrappers have been submitted to the Intergalactic Utilities Commission (IUC) tool repository [24] and are available from the Galaxy Tool Shed [25]. The IUC is a group of community members dedicated to developing and upholding Galaxy tool development best practices and guidelines, thus by contributing our tools to this repo we ensure that the tools will be well-maintained. A metagenomics Galaxy flavour [26] is available which contains all components presented here. The full mothur suite has also been installed to Galaxy's main server. [27].

KRONA visualization

KRONA [6] is a data viewer which provides the ability to interactively explore hierarchical data. A Galaxy KRONA wrapper that works directly on mothur data formats was developed for this project.

Phinch visualization

Galaxy offers integration with Phinch [16] BIOM format viewer in two ways; as a Galaxy interactive environment (GIE) developed in the context of this project [17], and more recently also as an external display application hosted by the Galaxy team.

iReport summarization

To facilitate the evaluation of 16S rRNA gene sequencing analysis results, integration with the iReport [22] tool are also provided. This tool creates a web report to present the analysis results in an organized fashion and provides links to external resources such as BLAST searches (Figure 2).

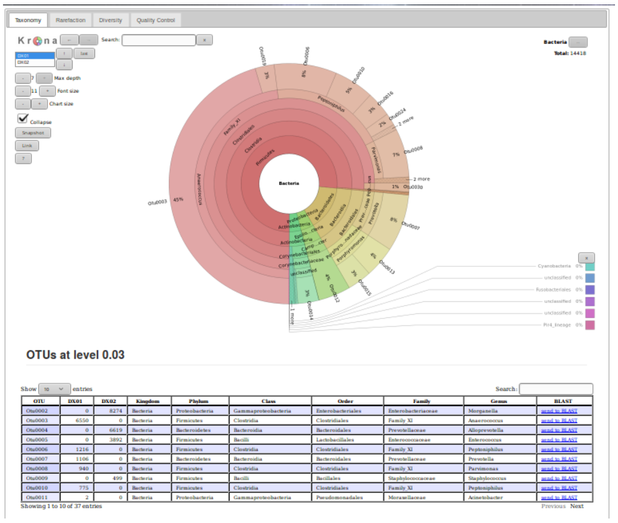

Figure 2. Example iReport. This web report contains the interactive KRONA visualization, the (multi-sample) OTU table, rarefaction plots, diversity calculations, differential abundance analysis, and an extensive overview of the quality control measurements taken during the analysis. iReports are highly customizable and can be easily tailored to fit specific use-cases and end-user needs.

Availability of source code and requirements

- Project name: Galaxy mothur Toolset (GMT)
- Project home page: https://github.com/erasmusmc-bioinformatics/galaxy-mothur-toolset
- Toolshed repository: https://toolshed.g2.bx.psu.edu/view/iuc/suite_mothur/768c2e48b706
- Training manual: https://galaxyproject.github.io/training-material
- GmT Docker image: https://quay.io/shiltemann/galaxy-mothur-toolset:16.07
- Galaxy Metagenomics Docker Flavour (Docker): https://quay.io/repository/shiltemann/galaxy-metagenomics, https://github.com/shiltemann/galaxy-metagenomics
- Phinch interactive environment: https://github.com/shiltemann/phinch-galaxy-ie
- Operating system(s): Unix (Platform independent with Docker)
- License: GNU GPL v3

Availability of supporting data and materials

The data presented here to illustrate our work is the same data used in the training manual, and is available from Zenodo [28]

Declarations

List of abbreviations

- GIE: Galaxy interactive environment
- GmT: Galaxy mothur Toolset
- GTN: Galaxy Training Network
- GUI: Graphical user interface
- SOP: Standard Operating Procedure

Competing Interests

The authors declare that they have no competing interests.

Funding

This work has received funding from the European Union's Seventh Framework Programme for Health under grant agreement number 602860 (TAILORED-Treatment; www.tailored-treatment.eu).

Author's Contributions

SH developed the Galaxy tool wrappers and Phinch interactive environment. SB validated the analysis pipelines. All authors contributed to the manuscript text and approve its contents.

Acknowledgements

The authors would like to thank Jim Johnson and the many other contributors and reviewers of the mothur tool wrappers, including everybody who contributed to the development of these tools within the context of the Galaxy metagenomics contribution fest organized by the Galaxy community's Intergalactic Utilities Commission (IUC), a group of community members dedicated to developing and upholding Galaxy tool development best practices and guidelines [29]. We would also like to thank the Galaxy Training Network for providing the infrastructure and valuable feedback to share our training materials.

References

1. Schloss PD, Westcott SL, Ryabin T, Hall JR, Hartmann M, Hollister EB, et al. Introducing mothur: open-source, platform-independent, community-supported software for describing and comparing microbial communities. Applied and environmental microbiology 2009;75(23):7537–7541.
2. Caporaso JG, Kuczynski J, Stombaugh J, Bittinger K, Bushman FD, Costello EK, et al. QIIME allows analysis of high-throughput community sequencing data. Nature methods 2010;7(5):335–336.
3. Glass EM, Wilkening J, Wilke A, Antonopoulos D, Meyer F. Using the metagenomics RAST server (MG-RAST) for analyzing shotgun metagenomes. Cold Spring Harbor Protocols 2010;2010(1):pdb–prot5368.
4. Oulas A, Pavloudi C, Polymenakou P, Pavlopoulos GA, Papanikolaou N, Kotoulas G, et al. Metagenomics: tools and insights for analyzing next-generation sequencing data derived from biodiversity studies. Bioinformatics and Biology insights 2015;9:75.
5. Afgan E, Baker D, Van den Beek M, Blankenberg D, Bouvier D, Čech M, et al. The Galaxy platform for accessible, reproducible and collaborative biomedical analyses: 2016 update. Nucleic acids research 2016;44(W1):W3–W10.
6. Ondov BD, Bergman NH, Phillippy AM. Interactive metagenomic visualization in a Web browser. BMC bioinformatics 2011;12(1):385.
7. Caporaso JG, Bittinger K, Bushman FD, DeSantis TZ, Andersen GL, Knight R. PyNAST: a flexible tool for aligning sequences to a template alignment. Bioinformatics 2009;26(2):266–267.
8. Langille MG, Zaneveld J, Caporaso JG, McDonald D, Knights D, Reyes JA, et al. Predictive functional profiling of microbial communities using 16S rRNA marker gene sequences. Nature biotechnology 2013;31(9):814.
9. Wood DE, Salzberg SL. Kraken: ultrafast metagenomic sequence classification using exact alignments. Genome biology 2014;15(3):R46.
10. Truong DT, Franzosa EA, Tickle TL, Scholz M, Weingart G, Pasolli E, et al. MetaPhlAn2 for enhanced metagenomic taxonomic profiling. Nature methods 2015;12(10):902.
11. Abubucker S, Segata N, Goll J, Schubert AM, Izard J, Cantarel BL, et al. Metabolic reconstruction for metagenomic data and its application to the human microbiome. PLoS computational biology 2012;8(6):e1002358.
12. Schmieder R, Edwards R. Quality control and preprocessing of metagenomic datasets. Bioinformatics 2011;27(6):863–864.
13. Rodriguez-r LM, Konstantinidis KT. Nonpareil: a redundancy-based approach to assess the level of coverage in metagenomic datasets. Bioinformatics 2013;30(5):629–635.
14. Dixon P. VEGAN, a package of R functions for community ecology. Journal of Vegetation Science 2003;14(6):927–930.
15. Eric R, Bjorn G, John C, Dannon B. Galaxy Interactive Environments–a new way to interact with your data. In: Galaxy Community Conference; 2015. .
16. Bik HM, Interactive P. Phinch: An interactive, exploratory data visualization framework for–Omic datasets. bioRxiv 2014;p. 009944.
17. Hiltemann S, Phinch Galaxy Interactive Environment; 2016. https://github.com/shiltemann/phinch-galaxy-ie.
18. The Biological Observation Matrix (BIOM) format;. http://biom-format.org/.
19. GTN Training Materials;. https://training.galaxyproject.org.
20. Training materials for using GmT to run the mothur miseq SOP;. https://galaxyproject.github.io/training-material//topics/metagenomics/tutorials/mothur-miseq-sop/tutorial.html.
21. Batut B, Gravouil K, Defois C, Hiltemann S, Brugère JF, Peyretaillade E, et al. ASaiM: a Galaxy-based framework to analyze raw shotgun data from microbiota. bioRxiv 2017;p. 183970.
22. Hiltemann S, Hoogstrate Y, Van Der Spek P, Jenster G, Stubbs A. iReport: a generalised Galaxy solution for integrated experimental reporting. GigaScience 2014;3(1):19.
23. Wilkinson MD, Dumontier M, Aalbersberg IJ, Appleton G, Axton M, Baak A, et al. The FAIR Guiding Principles for scientific data management and stewardship. Scientific data 2016;3:160018.
24. IUC tool repository;. https://github.com/galaxyproject/tools-iuc.
25. Galaxy Tool Shed;. https://toolshed.g2.bx.psu.edu/.
26. Metagenomics Galaxy Flavour;. https://github.com/shiltemann/galaxy-metagenomics.
27. Galaxy Main server;. https://usegalay.org.
28. Mothur MiSeq SOP Galaxy Tutorial Data;. https://zenodo.org/record/800651.
29. Intergalactic Utilities Commission;. https://galaxyproject.org/iuc/.

Click here to access/download
Supplementary Material
renamed_e6bce.pdf
