# Supplementary material for: Galaxy mothur Toolset (GmT): a user-friendly application for 16S rRNA gene sequencing analysis using mothur
Source: Gigascience. 2018 Feb 28;8(2):giy166. doi: 10.1093/gigascience/giy166 (PMC6377400; doi:10.1093/gigascience/giy166)
Supplement: Supplemental Files [file giy166_supplemental_files.zip › supplementary.pdf]

Supplementary Material

S1: Downstream Analysis Tools

To illustrate the utility of our tool suite, we have tested interoperability with downstream analysis and present some examples of tools previously wrapped for Galaxy our GmT tool suite is able to integrate with.

General interoperability features

In order to facilitate the interoperability of data generated with the mothur toolset and other Galaxy components we have completed the following:

1. We updated the Galaxy code base itself to include biom datatype definitions and automatic conversion tools

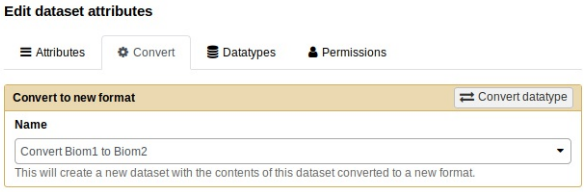

2. We created custom format conversion Galaxy tools such as taxonomy2krona which will take a mothur-formatted taxonomy file and reformat it to input ready for Krona tool. This facilitates connections with downstream analysis tools.

3. We integrated Phinch into Galaxy
a. We created the Phinch GIE (Galaxy Interactive Environment) to allow the running of Phinch within Galaxy itself (using docker containers)
b. For Galaxy instances not willing or able to support GIE's, link-outs to an existing Phinch server are available directly on the Galaxy history dataset item.

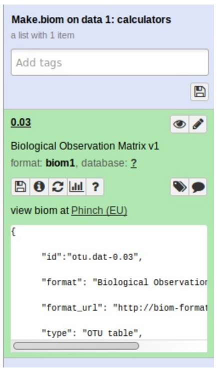

Downstream Visualisation and Analysis tools

Krona

Description

Krona allows hierarchical data to be explored with zooming, and multi-layered pie charts

Example

Below is an example Krona plot made with the example data provided with the mothur SOP (Standard Operating Procedure) described by the mothur developers team:

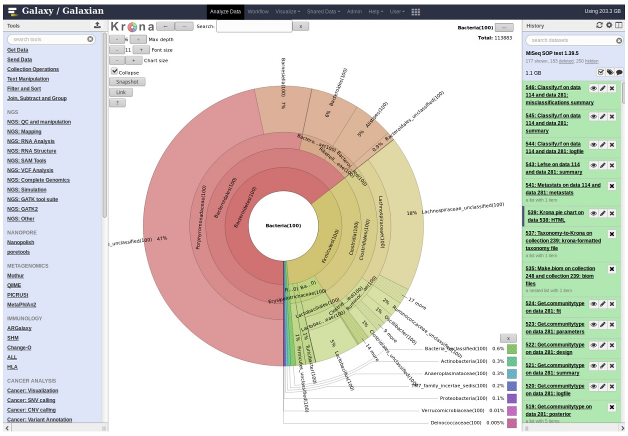

Steps necessary to connect

Use the conversion tool we developed as part of GmT named `taxonomy2krona` to convert a mothur-formatted OTU table to a format accepted by the Krona Galaxy tool.

Phinch

Description

Phinch provides an interactive visualization tool that allows users to explore and manipulate large biological datasets. We developed a Phinch GIE (Galaxy Interactive Environment) that enables researches to view their data in Phinch without leaving Galaxy.

Example

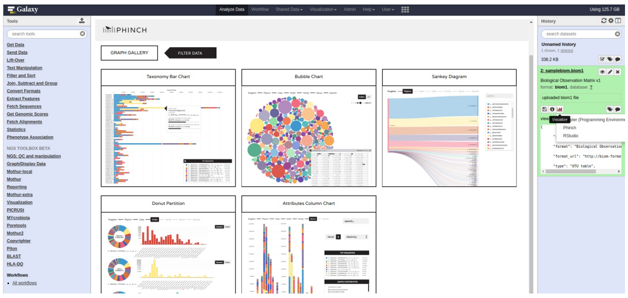

Steps necessary to connect

The output from the mothur `make.biom` tool will be accepted by Phinch without further modification.

- Use the mothur `make.biom` tool to create a biom file from your mothur result files
- Option 1: In the visualisations menu you will find an option to visualize with Phinch (GIE)
- Option 2: On the expanded dataset you fill find an option to visualize with Phinch (external server)

Vegan

Description

The vegan package provides tools for descriptive community ecology. It has most basic functions of diversity analysis, community ordination and dissimilarity analysis. Most of its multivariate tools can be used for other data types as well.

Tools

Vegan is an R package, and three different Galaxy tools are available based on this R package:

- Vegan Rarefaction
- Vegan Fisher Alpha index
- Vegan Diversity index

Example

Below is an example of a rarefaction plot made using the Galaxy Vegan tool:

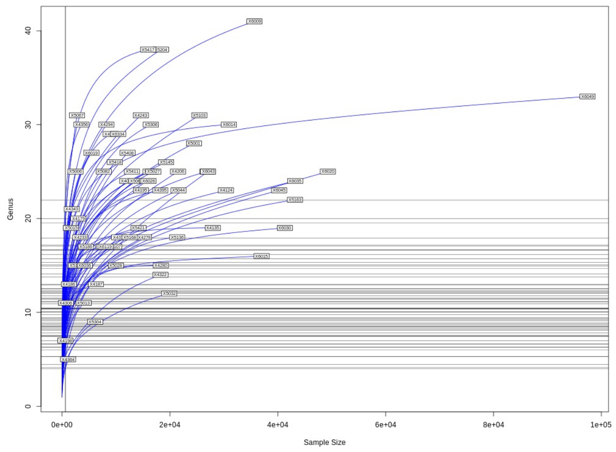

Steps necessary to connect

- A mothur-formatted *shared* file can be input into this tool without modification
- Choose column 1 as sample name column

- Choose all other columns except column 3 as data colum

GitHub: https://github.com/vegandevs/vegan/

Qiime

Description

QIIME is an open-source bioinformatics pipeline for performing microbiome analysis from raw DNA sequencing data. QIIME is designed to take users from raw sequencing data generated on the Illumina or other platforms through publication quality graphics and statistics. This includes demultiplexing and quality filtering, OTU picking, taxonomic assignment, and phylogenetic reconstruction, and diversity analyses and visualizations. QIIME has been applied to studies based on billions of sequences from tens of thousands of samples.

Tools

Currently 34 of Qiime's analysis commands are wrapped in Galaxy. Most of these require just a biom file as input and are compatible with mothur-generated biom files.

Example

As an example, below is a heatmap made using qiime in Galaxy (tool `make_otu_heatmap`) on a biom file generated from GmT analysis run.

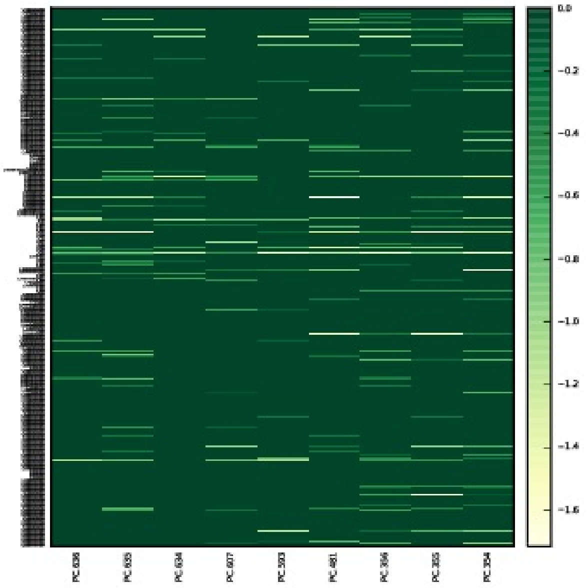

Steps to connect

Qiime works on biom files (hdf-5 format).

- Use the mothur command `make.biom` in Galaxy to create a biom file from a mothur OTU table or shared file

- Use the converter we contributed to Galaxy to convert this to biom2 (hdf5) format

Newick display tool

Description

This is a tool in Galaxy that will perform operations on Newick trees. The Newick Utilities are a set of tools for working with phylogenetic trees. They are not tools for making phylogenies. Rather, they are for processing existing ones, for example manipulating the tree or extracting information from it; displaying, rerooting, simplifying, extracting subtrees, printing branch lengths and distances, etc. We have created Galaxy tool wrappers under the IUC (Intergalactic Utilities Commission) umbrella for the Newich display tool.

Example

Below is an example of a Newick tree display generated within Galaxy on mothur-generated data using this tool.

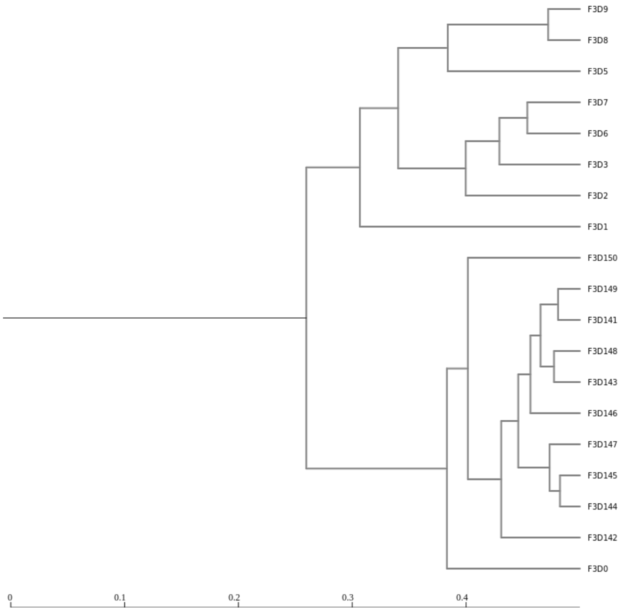

Steps to connect

- Use the output from GmT mothur wrapper `make.tree` directly as input for the Newick display tool

PICRUSt

Description

The PICRUSt project aims to support prediction of the unobserved character states in a community of organisms from phylogenetic information about the organisms in that community. The primary application is to predict gene family abundance (e.g. the metagenome) in environmental DNA samples for which only marker gene (e.g. 16S rRNA gene) data are available.

This is an open source, international, collaborative bioinformatics project developed in the Huttenhower, Beiko, Langille, Vega Thurber, Knight and Caporaso labs.

Tools

PICRUSt consists of a number of different commands, which each have
been made available in Galaxy:

- Metagenome Contributions
- Format tree and trait tables
- Compare BIOM tables
- Predict Metagenome - based on the abundance of OTUs and a functional database
- Categorize - collapses hierarchical data to a specified level

Steps to connect

- Output of the GmT mothur wrapper `make.biom` can be input into PICRUSt directly

S2: Benchmarking

To show the utility of this tool set, we have run a number of analyses on different sample sizes trough the Mothur SOP pipeline and report here the total wall time between start and finish. Please note that this is only an example, and results will vary significantly depending on a number of factors:

1) **Compute resources** underlying the Galaxy instance of your choice. Galaxy is not a single service. Anybody can run their own Galaxy instance and there are currently over 100 public instances and many more private instances where research or diagnostics group run their own Galaxy instance on dedicated compute resources and directly connected to data storage to prevent the need for data transfer.

2) **Length of the Galaxy queue** times at the time of analysis. When the Galaxy instance is shared with many users, job will be managed by a job scheduler and will have to wait in a queue for their turn to arrive and sufficient resources required by the job to be available. Queue times will vary greatly between different Galaxy servers and at different times of the day/week/year.

3) **The connection speed** between source of the input files and the Galaxy instance. Many researchers will run Galaxy close to their data storage to minimize data transfer times. Transferring data across slow connections will of course increase the upload times.

4) **Method of file transfer** For large files or sets of files, FTP transfer can be utilized which will have differing upload speeds to the case where data is transferred via the web interface.

This benchmarking was performed on our local Galaxy server, which has relatively low queue times. For the purposes of this benchmark we configured the tools to use only a single core as to

represent a worst-case time frame. We also ran the workflows on 16 cores, which provided a significant speedup. In practice, run times will often be limited by the amount of time spent waiting in the job queue. No conclusions can be drawn from these results about analysis or upload times on different Galaxy instances.

Samples	Reads	Upload time	Runtime (1 core)*	Runtime (16 cores)*
2	25,144	00:00:06	00:02:48	00:01:03
20	304,720	00:00:46	00:10:03	00:04:57
188	3,533,776	00:13:07	02:01:43	00:22:07
362	7,303,568	00:22:19	03:53:34	00:51:56

* Run times are including all time spent waiting in the job queue.
