# Supplementary material for: Galaxy mothur Toolset (GmT): a user-friendly application for 16S rRNA gene sequencing analysis using mothur
Source: Gigascience. 2018 Feb 28;8(2):giy166. doi: 10.1093/gigascience/giy166 (PMC6377400; doi:10.1093/gigascience/giy166)
Supplement: Response_to_Reviewer_Comments_Original_Submission.pdf [file giy166_response_to_reviewer_comments_original_submission.pdf]

# Author's Response To Reviewer Comments

Close

Dear Editorial board and reviewers,

Thank you for the opportunity to submit a revision, and thank you for your comments and insight. We have addressed all reviewers' concerns, and have outlined a detailed response in the file response-to-reviewers.pdf included in this submission.

Best regards,

Saskia

Close
