# Supplementary material for: Galaxy mothur Toolset (GmT): a user-friendly application for 16S rRNA gene sequencing analysis using mothur
Source: Gigascience. 2018 Feb 28;8(2):giy166. doi: 10.1093/gigascience/giy166 (PMC6377400; doi:10.1093/gigascience/giy166)
Supplement: Response_to_Reviewer_Comments_Revision_1.pdf [file giy166_response_to_reviewer_comments_revision_1.pdf]

Author's Response To Reviewer Comments

Close

We have added the link to the GigaDB entry. Thanks for all your help in getting this prepared.

Close
