# Supplementary material for: Galaxy mothur Toolset (GmT): a user-friendly application for 16S rRNA gene sequencing analysis using mothur
Source: Gigascience. 2018 Feb 28;8(2):giy166. doi: 10.1093/gigascience/giy166 (PMC6377400; doi:10.1093/gigascience/giy166)
Supplement: Reviewer_2_Report_original_submission -- Xianfeng Chen — 4/16/2018 Reviewed [file giy166_reviewer_2_report_original_submission.pdf]

Reviewer Report

Title: Galaxy mothur Toolset (GmT): a user-friendly application for 16S rRNA gene sequencing analysis using mothur.

Version: Original Submission **Date:** 4/16/2018

Reviewer name: Xianfeng Chen

Reviewer Comments to Author:

The revised version has addressed some of my concerns including a little more introductions about Galaxy, package installations, large file transfer, etc. Yet, it could not completely convince me without a demonstration how robust this tool is, a plot of running time vs. the number of total reads (starting from uploading fastq files) may help as described in 1.b in my previous comments.
Or, the authors can emphasize the novelty by, for example, involving more downstream analysis tools to make this tool a upgrade version of mothur as described in the last paragraph of my previous comments.

Level of Interest

Please indicate how interesting you found the manuscript: Choose an item.

Quality of Written English

Please indicate the quality of language in the manuscript: Choose an item.

Declaration of Competing Interests

Please complete a declaration of competing interests, considering the following questions:

- Have you in the past five years received reimbursements, fees, funding, or salary from an organisation that may in any way gain or lose financially from the publication of this manuscript, either now or in the future?
- Do you hold any stocks or shares in an organisation that may in any way gain or lose financially from the publication of this manuscript, either now or in the future?
- Do you hold or are you currently applying for any patents relating to the content of the manuscript?
- Have you received reimbursements, fees, funding, or salary from an organization that holds or has applied for patents relating to the content of the manuscript?
- Do you have any other financial competing interests?
- Do you have any non-financial competing interests in relation to this paper?

If you can answer no to all of the above, write 'I declare that I have no competing interests' below. If your reply is yes to any, please give details below.

I declare that I have no competing interests' below. If your reply is yes to any, please give details below

I agree to the open peer review policy of the journal. I understand that my name will be included on my report to the authors and, if the manuscript is accepted for publication, my named report including any attachments I upload will be posted on the website along with the authors' responses. I agree for my report to be made available under an Open Access Creative Commons CC-BY license (http://creativecommons.org/licenses/by/4.0/). I understand that any comments which I do not wish to be included in my named report can be included as confidential comments to the editors, which will not be published.

I agree to the open peer review policy of the journal
