# Supplementary material for: Galaxy mothur Toolset (GmT): a user-friendly application for 16S rRNA gene sequencing analysis using mothur
Source: Gigascience. 2018 Feb 28;8(2):giy166. doi: 10.1093/gigascience/giy166 (PMC6377400; doi:10.1093/gigascience/giy166)
Supplement: Supplemental Files [file response-to-reviewers.pdf › response-to-reviewers.pdf]

Response to Reviewers

Thank you for your comments, and we have addressed all the remaining remarks as explained in the following sections. First, to address the reviewers' concerns about downstream analysis options, we now outline various possibilities for downstream analysis tools more clearly in a supplementary data section. Secondly, with regards to the concern about feasibility and utility of this Galaxy approach in terms of upload times and workflow run times, we have performed a benchmark on datasets of different sizes and report upload and compute times for each.

Downstream analysis options

To address comments about interoperability of our tool suite with downstream analysis packges, we have highlighted some of these possible downstream tools more prominently in a new supplementary materials section; we describe a number of tools, which file conversion steps or parameter settings are required for those tools to work with mothur output formats, and give example outputs in the case of visualisation tools.

In order to facilitate interoperability with downstream analysis tools, we have also contributed to the Galaxy core code in the form of datatype definitions, both for mothur-specific formats, as for the more general biom format (both the json and hdf5 versions), and have added converters between them to the Galaxy user interface (see this pull request) and the supplementary material for more details). Furthermore, we have created custom Galaxy tools, such as `taxonomy2krona` to convert mothur formatted output to specific formats required by downstream analysis tools (in this example Krona).

Because mothur has its emphasis more on data analysis than visualisation, we have developed and tested several downstream visualisation options.

1. We created a Phinch GIE (Galaxy interactive environment) so that users may view their biom files in Phinch directly within Galaxy. (see pull request)
2. We have contributed wrappers to the IUC Galaxy repository for visualisation of Newick trees, an output which mothur also produces. (pull request)
3. We implemented PICRUSt wrappers into Galaxy within the IUC repository. (pull request)
4. We have co-contributed to the Qiime wrappers, many of which offer visualisation based on biom files, an output mothur produces.
5. We verified integration with the Vegan Galaxy tools

6. We tested integration with a number of the more generic visualisation tools in Galaxy and contributed modifications where it streamlined integration with mothur-generated outputs (example pull request).

The list of downstream tools our toolset is able to work with in Galaxy include (but not limited to): Krona, Phinch, Qiime, PiCRUST, Vegan, Newick display, XY_plot, Venn diagram plotters, and heatmap tools. And because mothur outputs several standard output formats (biom files, newick trees), there are likely many more possible downstream tools in the Galaxy toolshed since submitting this article, and will continue to grow in the future.

Many of these tools and features were developed or co-developed by us and have been contributed to the Galaxy community via the IUC tool repository or directly in the Galaxy core code base. Galaxy is a continually evolving community, and the list of downstream tools will grow annually, both through continued contributions from us, as by contributions of the rest of the community.

Benchmarking

To address concerns about feasibility and utility of running these kinds of analyses within the Galaxy framework, we have performed a benchmark on datasets of different sizes running the MiSeq SOP as defined by the mothur development team. We have added a table with the results to the supplementary materials section, and report both upload times and compute times, both with Galaxy configured to use 1 core and 16 core backends. These results will vary greatly depending on the Galaxy server of choice, the length of the job queue and the time of day, and the user's internet connection for the upload times, but we hope that these benchmarks give at least an indication that processing of large datasets is feasible.

General remarks

To further illustrate the utility of our tools, I would like to point out that these tools have already been installed to both the Galaxy main instance (https://usegalaxy.org) and Galaxy EU (https://usegalaxy.eu); the latter of which also provides usage statistics of tools. Below is a plot of the average usages of these wrappers. As you can see, these tools are currently being used at a rate of 3K-5K jobs per month (link).

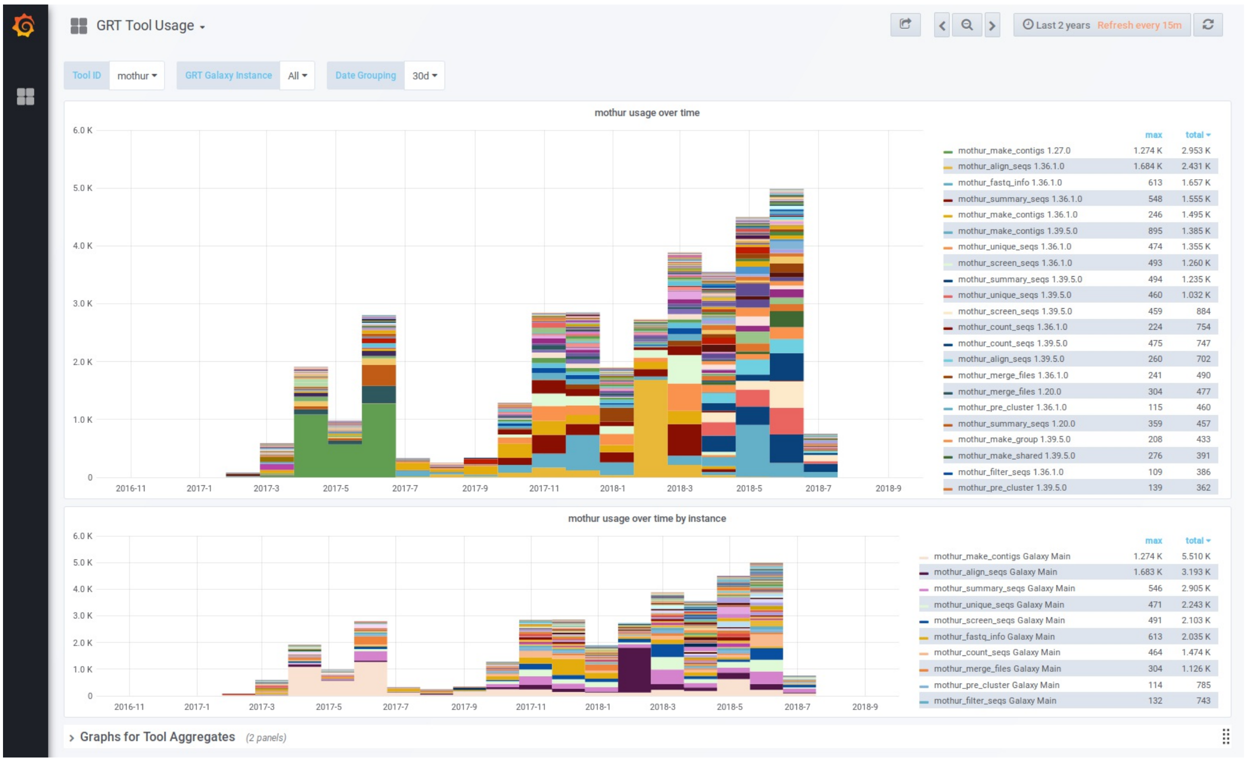

Furthermore, to address concerns about the maintenance of these tools; since the first submission we have already performed one major version upgrade of our tools (1.36.1 to 1.39.5) and have made preparations for the upgrade to version 1.41 once mothur developers release that version officially (currently in pre-release). We have received contributions from many community members to our tools, and are confident that we can keep these tools maintained as a community.
